# Exploring the COVID-19 pandemic’s influence on eating disorders: insights from an Italian residential center

**DOI:** 10.3389/fpsyg.2025.1522599

**Published:** 2025-03-26

**Authors:** Francesco Monaco, Annarita Vignapiano, Paolo Meneguzzo, Stefania Palermo, Annaluce Caputo, Ilona Forte, Antonella Boccia, Anna Longobardi, Marilena Di Pierro, Ernesta Panarello, Benedetta Di Gruttola, Annarita Mainardi, Rossella Bonifacio, Emanuela Ferrara, Alessandra Marenna, Martina Piacente, Stefania Landi, Mauro Cozzolino, Luca Steardo Jr, Giulio Corrivetti

**Affiliations:** ^1^Department of Mental Health, ASL Salerno, Salerno, Italy; ^2^European Biomedical Research Institute of Salerno (EBRIS), Salerno, Italy; ^3^Department of Neuroscience, University of Padua, Padua, Italy; ^4^Department of Human, Philosophical and Educational Sciences, University of Salerno, Fisciano, SA, Italy; ^5^Department of Health Sciences, University Magna Graecia of Catanzaro, Catanzaro, Italy

**Keywords:** COVID-19, eating disorders, Anorexia Nervosa, Bulimia Nervosa, mental health, retrospective analysis, pandemic impact

## Abstract

**Introduction:**

The COVID-19 pandemic has significantly impacted global mental health, exacerbating the prevalence and severity of Eating Disorders (EDs). This study evaluates changes in the presentation and severity of EDs before and after the pandemic at the Regional Residential Center “Mariconda” in Salerno.

**Methods:**

This retrospective cohort study analyzed records from 162 patients admitted to the center between December 2018 and December 2023. The onset of pandemic restrictions in mid-2020 divided the subjects into pre-COVID and COVID groups. Data collected included age, gender, education level, previous hospital admissions, admission diagnoses, body mass index (BMI), and comorbidity with other psychiatric conditions.

**Results:**

Among the 162 subjects, 115 (71%) were admitted during the pandemic period. This group was significantly younger (mean age: 18.3 vs. 20.6 years, *p* = 0.009), had lower educational attainment (67% vs. 49% below secondary school diploma, *p* = 0.025), had a higher rate of prior hospitalizations (49% vs. 26%, *p* = 0.007), and demonstrated a higher prevalence of severe comorbidities with other mental illnesses (94% vs. 82%, *p* = 0.009) compared to the pre-COVID cohort. No significant differences were observed in gender distribution, initial diagnoses upon admission, or average length of hospital stay.

**Conclusion:**

The findings indicate that the COVID-19 pandemic intensified the presentation and severity of EDs, particularly among younger individuals with lower educational backgrounds. This underscores the urgent need for targeted, integrated treatment approaches for EDs in the context of global crises, including the development of strategies to address the increased severity of comorbid conditions and higher frequency of hospital readmissions observed in this study. These results highlight the necessity of reinforcing multidisciplinary care models that integrate medical, psychological, and social support to address the heightened complexity of post-pandemic ED cases and ensure more effective, long-term treatment outcomes. Future research is essential to explore the long-term effects of the pandemic on EDs, as well as to refine treatment strategies that better support those affected.

## Introduction

The COVID-19 pandemic has profoundly disrupted global health systems and significantly exacerbated existing mental health challenges, particularly Eating Disorders (EDs) (Monteleone, 2021; Meier et al., 2022). Even before the pandemic, eating disorders affected a substantial portion of the global population, with varying prevalence rates across different demographics (Monteleone, 2021).

EDs, including Anorexia Nervosa (AN), Bulimia Nervosa (BN), and Binge Eating Disorders (BED), are marked by severe disturbances in eating behavior and unhealthy preoccupations with body weight and shape (Zipfel et al., 2015; McLean et al., 2022). These disorders can lead to critical physical and psychological consequences.

While pre-pandemic eating disorders presented a notable yet often under-recognized global health challenge, the COVID-19 pandemic increased existing vulnerabilities and revealed critical gaps in ED care. Specifically, the pandemic’s disruption of routine healthcare, increased social isolation, and heightened anxiety surrounding food and body image amplified ED risk and hindered access to timely and effective treatment (Monteleone, 2021; McLean et al., 2022; Monteleone et al., 2021). A 2022 review found that the prevalence of eating disorders worldwide increased during the pandemic, with a sharp rise in cases among adolescents and young adults (McLean et al., 2022).

The pandemic amplified these issues through stringent social restrictions and disruptions to daily routines, which heightened anxiety and stress levels across populations (Weissman et al., 2020; Monteleone et al., 2021). Younger individuals may be disproportionately affected by the pandemic’s impact on EDs due to several factors. School closures and social distancing measures disrupted established routines and support systems crucial for their development and well-being, leading to increased social isolation, heightened anxiety, and reduced access to regular healthcare (Weissman et al., 2020; Monteleone et al., 2021). This exacerbated existing vulnerabilities and triggered the onset or worsening of EDs, particularly for those already prone to disordered eating patterns. The loss of structured routines, and especially the predictable school schedule, proved particularly harmful, often resulting in worsening symptoms and increased relapse rates among individuals with EDs (Jeffers et al., 2022; Mentzelou et al., 2024). Adolescents and young adults were particularly vulnerable due to the abrupt disruption of their daily routines, the loss of structured environments such as school, and increased social isolation. School closures limit access to in-person social support from peers, teachers, and counselors, which are critical protective factors against mental health deterioration. Additionally, greater exposure to social media and online content during lockdowns may have heightened body image concerns and disordered eating behaviors. Reduced access to healthcare services, including early intervention and outpatient support, further compounded these risks, contributing to the observed increase in ED severity and prevalence in this age group (Benner et al., 2025; Garagiola et al., 2022; Ng and Ng, 2022).

Studies from various regions have documented the pandemic’s clinical impact, emphasizing how lockdowns have altered the presentation of EDs (Güzel et al., 2023; Meneguzzo et al., 2023). Individuals with EDs not only faced more severe repercussions than the general population or those with specific vulnerabilities (Castellini et al., 2020) but also experienced greater exacerbation of symptoms, presenting high rates of depression and anxiety (Linardon et al., 2021). Furthermore, even individuals without pre-existing diagnoses were at risk, as pandemic-related stressors increased maladaptive coping strategies such as emotional eating (Koithan et al., 2023). These findings highlight the urgent need for targeted interventions and deeper investigations into the unique effects of the pandemic on EDs, as existing literature has yet to reach conclusive answers (Güzel et al., 2023; Devoe et al., 2022). Additionally, the Campania region, where this study was conducted, was among the Italian regions that adopted some of the most restrictive measures to prevent the spread of COVID-19. During the pandemic, drastic public health interventions including strict lockdowns, curfews, and prolonged social distancing policies were enforced. These restrictions, while essential in controlling viral transmission, may have further exacerbated the impact of the pandemic on individuals with EDs, limiting their access to healthcare services, disrupting treatment continuity, and intensifying social isolation. Understanding how these region-specific policies influenced ED severity and treatment demand is crucial for developing effective, adaptive care models in future crises.

### Aims of the project

This study aims to assess the changes in the presentation and severity of EDs before and after the pandemic at the Regional Residential Center for Eating Disorders “Mariconda” in Salerno. The findings aim to offer critical insights that will increase the currently available information about the effect of the pandemic on the ED field, looking at the possible development of more effective treatment strategies and interventions, ensuring that individuals with EDs receive the necessary support and care in future situations of global crisis.

## Methods

This study adopted a retrospective cohort design, leveraging pre-existing data from the “Mariconda” Residential Center for Eating Disorders. The dataset was selected as it spans a five-year period (December 2018–December 2023), enabling a comprehensive comparison between pre-pandemic and pandemic periods. The implementation of pandemic restrictions in mid-2020 in the Campania region served as a natural dividing line, creating two comparable groups: those admitted before and those admitted during the pandemic. This approach allowed researchers to assess the impact of the pandemic on ED characteristics without the need for prospective data collection.

The inclusion criteria required patients to have been admitted to the center within the designated timeframe and to have a clinically diagnosed ED according to DSM-5-TR criteria, as determined by a trained psychiatrist through a clinical interview based on the Structured Clinical Interview for DSM-5-TR (SCID-5-CV) (American Psychiatric Association, 2013). Additionally, patients had to be at least 13 years old, in accordance with the Center’s Access Regulations. For minor patients, informed consent was obtained from their parents or legal guardians. Eligible ED diagnoses included AN, BN, and BED.

Comprehensive sociodemographic and clinical data were systematically collected from medical records, which included detailed patient histories. Additionally, upon admission, each patient underwent a thorough clinical and psychopathological assessment to evaluate psychiatric comorbidities. The collected variables included age, gender, educational background, history of prior hospitalizations, admission diagnosis, body mass index (BMI), psychiatric comorbidities (such as psychotic features) and, where applicable, the presence of amenorrhea. These data were subsequently entered into Google Sheets, a widely used cloud-based tool for data management, analysis, and collaboration.

**Figure 1:**
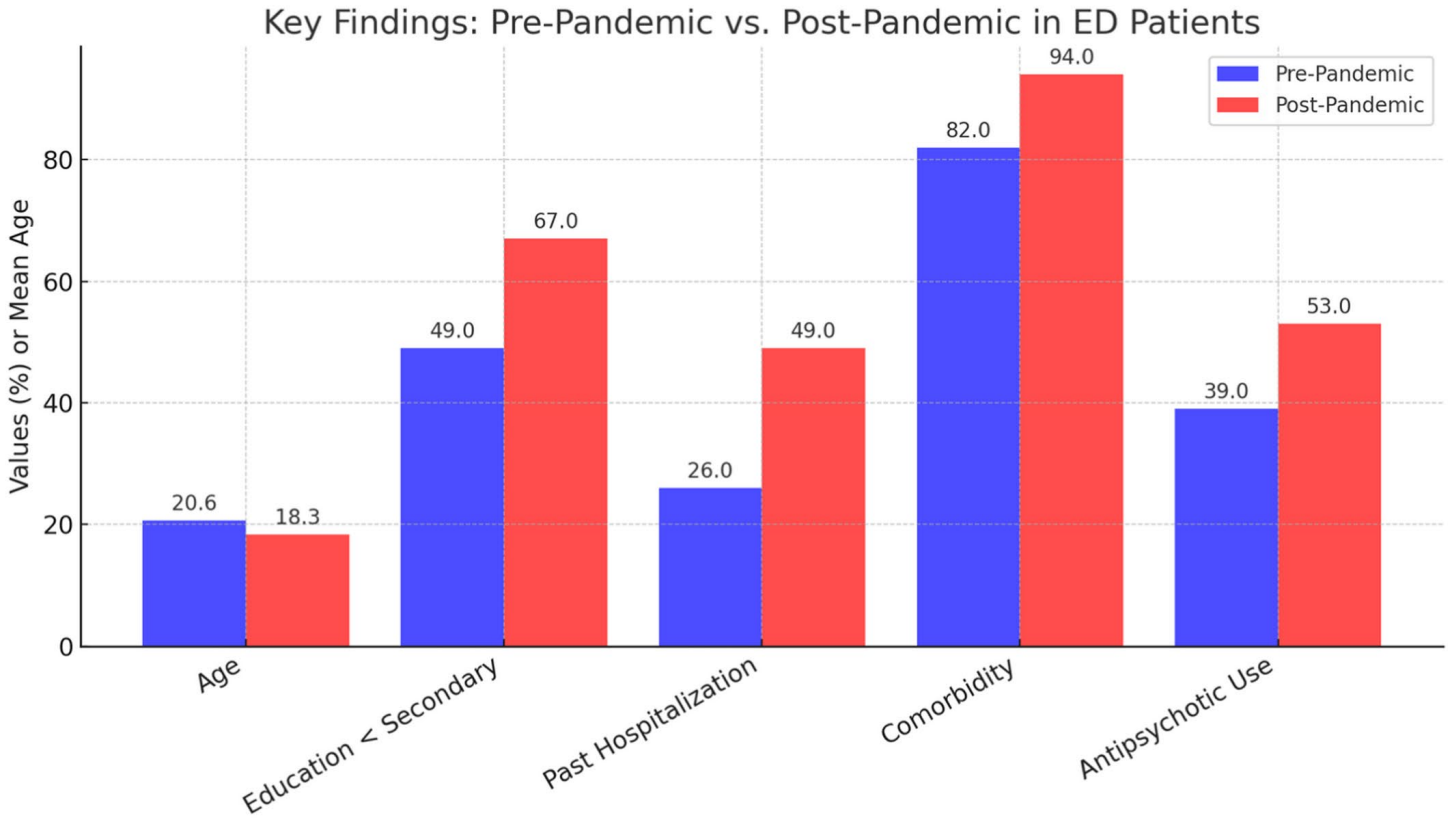
Overview of the analysed sample.

This detailed profiling allowed for a nuanced understanding of patient characteristics across both temporal groups. Patients were systematically categorized into pre-pandemic and pandemic groups based on their admission dates. This classification facilitated a clear comparison of how the pandemic and its associated restrictions influenced the presentation and progression of EDs, contributing to a deeper understanding of potential shifts in clinical manifestations and informing optimized treatment strategies (Figure 1).

### Statistical analysis

The statistical analysis in this study was conducted using standard inferential statistics to compare the pre-pandemic and post-pandemic groups across several demographic and clinical variables. Descriptive statistics, including means and standard deviations, were calculated for continuous variables such as age and BMI, while categorical variables like gender, educational attainment, and comorbidities were reported as percentages. For comparing the two groups, t-tests were used for continuous variables (e.g., age) to assess whether there were statistically significant differences between the pre- and post-pandemic cohorts. A chi-square test was employed for categorical variables (e.g., gender distribution, comorbidities) to evaluate whether the observed differences were due to chance. Statistical significance was set at a *p*-value of less than 0.05. Statistical analyses were carried out using the StatPlus software. StatPlus is a statistical analysis software designed for professionals, researchers, and students who need an efficient yet user-friendly tool for data processing, statistical analysis, and visualization. StatPlus is a practical and efficient solution for users who need robust statistical analysis without the complexity of coding-based software. Its combination of accessibility, powerful statistical functions, and Excel integration makes it a strong choice for professionals and researchers seeking an effective yet easy-to-use analytics tool.

## Results

### Demographic characteristics

This study analyzed 162 patients admitted to the Regional Residential Center for Eating Disorders “Mariconda” in Salerno, revealing a significant transformation in demographic and clinical characteristics between those admitted before and during the COVID-19 pandemic. In particular, 115 patients, or 71% of the total cohort, were admitted during the pandemic period, forming what is known as the pandemic group. This group exhibited a marked difference in age distribution, with a higher prevalence of younger individuals being admitted in the period following the onset of the pandemic. In addition to age differences, educational attainment emerged as another critical factor distinguishing the two groups. The pandemic cohort exhibited significantly lower levels of educational achievement compared to their pre-pandemic counterparts, which aligns with their younger ages. The *p*-values reported in Table 1 indicate that there were statistically significant differences between the pre- and post-pandemic groups in terms of age (*p* = 0.009), educational attainment (*p* = 0.025), previous hospitalizations (*p* = 0.007), comorbidities (*p* = 0.009), and the use of antipsychotic medications (*p* = 0.050).

**Table 1:** Demographic and clinical characteristics.

	Age	Education	Past hospitalization	Comorbidity	Antipsychotic
Pre-pandemic	20.6	±49% < secondary school diploma	26%	82%	39%
Post-pandemic	18.3	±67% < secondary school diploma	49%	94%	53%
*p*-value	0.009	0.025	0.007	0.009	0.050

### Clinical characteristics

Despite the differences in demographic characteristics, no significant differences were found between the pre-pandemic and pandemic groups regarding gender distribution, initial diagnosis upon admission, average BMI or the presence of amenorrhea. The distribution of ED diagnoses was as follows: 81% AN in pre-pandemic group vs. 89% AN in pandemic group; 17% BN in pre-pandemic group vs. 9% BN in pandemic group; 2% BED in pre-pandemic group vs. 2% BED in pandemic group.

### Comorbidities with other mental illnesses

During the post-pandemic period, patients admitted to our center exhibited more severe psychopathological profiles, highlighting the complex impact of the pandemic on mental health. Specifically, there was a marked increase in psychotic spectrum disorders requiring medication, estimated between 20 and 25%. This rise in comorbid psychosis may be linked to pandemic-related stressors, including social isolation, heightened anxiety, and disruptions to daily routines, which likely exacerbated pre-existing vulnerabilities.

### Hospital admission pattern

A noteworthy observation in this study was the significant rise in the frequency of previous hospital admissions among the post-pandemic group. This trend suggests that individuals admitted during the pandemic period were more likely to have experienced severe episodes of EDs. Despite the heightened frequency of admissions, the length of hospital stay did not significantly differ between the pre-pandemic and post-pandemic groups.

This finding may indicate that while the severity or recurrence of episodes increased, the treatment protocols and resources available remained effective in managing acute. It highlights the resilience and adaptability of the healthcare system in maintaining consistent care standards even amid a global crisis (Table 1).

## Discussion

The study’s findings reveal that the COVID-19 pandemic has markedly influenced the presentation and severity of EDs. The pandemic group was characterized by younger age and lower educational levels, suggesting that younger individuals may have been disproportionately impacted. Disruptions caused by the pandemic, including school closures and prolonged social isolation, likely contributed to the earlier onset and heightened severity of EDs in this demographic. These factors underscore the increased vulnerability of younger populations during such global crises and highlight the need for targeted interventions to mitigate these adverse effects (Irwin et al., 2021). The significantly higher rate of hospital admissions in the post-pandemic group, a 48% average increase overall and an 83% increase in pediatric admissions compared to pre-pandemic levels, strongly suggests increased severity and recurrence of eating disorder episodes during the pandemic (Devoe et al., 2022). This suggests that stress and uncertainty during this period, along with disruptions to regular healthcare services, may have resulted in delayed treatment and more severe presentations upon admission. The interplay between social isolation, loss of structure, and increased reliance on maladaptive coping mechanisms contributed to the worsening of ED severity. Many individuals, particularly younger patients, reported increased preoccupation with body image and food intake due to heightened exposure to social media, where unrealistic beauty standards and diet culture were amplified during lockdowns. The lack of access to in-person therapy and structured support further exacerbated these vulnerabilities, underlining the need for more proactive and adaptable intervention strategies. These findings align with previous studies that reported changes in the clinical presentation of individuals requiring specialized intensive treatment and support the idea that the pandemic may have significant implications for healthcare organizations (Todisco et al., 2023; Meneguzzo et al., 2023; Maina et al., 2023). Similar trends have been observed in other international studies, which reported a surge in ED severity, increased psychiatric comorbidities, and higher rates of hospitalization during the pandemic. For example, recent findings indicate that adolescents, particularly females, experienced greater distress due to school closures and social isolation, exacerbating disordered eating behaviors (Vitagliano et al., 2021). However, some studies suggest regional differences in ED presentation, potentially influenced by variations in healthcare accessibility, socioeconomic factors, and cultural attitudes toward mental health. Further research is needed to explore these contextual differences (Burnette et al., 2022). Moreover, the description of more severe symptomatology, with higher levels of psychotic symptoms, may highlight evidence of a significant and profound shift in the landscape of EDs reported worldwide (Castellini et al., 2020). Social isolation disrupted daily routines and coping mechanisms, leading to an increase in maladaptive behaviors such as binge eating and purging (Straface et al., 2023). This mirrors the study’s findings of increased psychotic symptoms, including hallucinations and paranoia, likely exacerbated by the pervasive anxiety and isolation brought on by the pandemic (Martini et al., 2023).

Also, the pandemic significantly exacerbated the mental health burden on individuals from lower socio-economic backgrounds (Fiorillo et al., 2016). This impact was largely driven by heightened stress levels, reduced access to healthcare services, and constrained financial resources. These challenges compounded the difficulties faced by these individuals, limiting their ability to seek timely and effective treatment and further destabilizing their mental well-being. This situation highlights the critical need for accessible mental health support and resources targeted at socio-economically disadvantaged populations, especially in the context of widespread crises. Napp et al. (2023) examined the disproportionate impact of the pandemic on younger individuals, particularly adolescents from low-income families, who experienced heightened levels of anxiety and depression. This emotional strain led to a worsening of ED symptoms, particularly among females, who reported higher anxiety levels compared to males. These observations line up with the findings of this study, which revealed that younger individuals with lower educational attainment exhibited more severe psychopathological profiles in the pandemic period (Todisco and Donini, 2020). This underscores the intersection of age, socio-economic status, and gender in influencing mental health outcomes during the pandemic, highlighting the urgent need for targeted interventions to support the most vulnerable groups. Similarly, Smith et al. (2024) found that families facing economic instability during the pandemic experienced heightened psychological distress, contributing to the onset or exacerbation of EDs. The financial strain and caregiver burden further limited these families’ access to timely mental health interventions, which contributed to an increase in comorbidities such as depression and psychosis. The data underscores the urgent need for targeted, comprehensive treatment strategies that address the unique challenges posed by the pandemic. Given the increasing complexity of ED cases, it is imperative to implement multidisciplinary interventions that integrate psychiatric, nutritional, and psychological care. Beyond reinforcing multidisciplinary treatment approaches, it is essential that healthcare policymakers develop crisis-response frameworks that ensure timely access to specialized ED care. This could involve the establishment of dedicated emergency ED units, expanding public health funding for outpatient and residential treatment programs, and integrating ED management strategies into broader pandemic preparedness plans. Additionally, the creation of targeted public awareness campaigns could help mitigate ED risk by promoting early intervention and reducing stigma around seeking mental health support. Moreover, the expansion of telehealth services, while beneficial during lockdowns, presents challenges for patients with severe EDs who require close clinical monitoring. Hybrid care models combining remote and in-person support may help bridge this gap, improving long-term treatment outcomes. Policymakers should prioritize funding for ED treatment programs, ensuring equitable access to specialized care, particularly for vulnerable populations disproportionately affected by the pandemic. Early intervention is particularly critical for younger individuals, who seem more vulnerable to pandemic-related effects (Bell et al., 2023). Integrated approaches combining medical, psychological, and nutritional support is essential to meet the complex needs of ED patients effectively (Pehlivan et al., 2022). This shift emphasizes the importance of comprehensive treatment plans that incorporate both pharmacological, psychotherapeutic and cognitive rehabilitative strategies, ensuring holistic care (Muratore and Attia, 2020). The increased complexity of cases observed during the pandemic has significant implications for healthcare systems. As highlighted in the findings of this study, the rise in antipsychotic use among the pandemic group underscores the need for more intensive, multidisciplinary treatment approaches. According to a 2024 report by STRIPED and Deloitte, the economic costs of EDs are not limited to direct treatment expenses but also include broader societal costs, such as loss of productivity and informal caregiving. This report stresses the importance of developing comprehensive care models that address both the medical and psychological aspects of EDs to reduce the long-term societal burden (Boltri et al., 2024). Additionally, it highlights the critical need for mental health services to adapt rapidly to emerging patterns in mental illness precipitated by global crises. Strengthening mental health services and support systems to handle increased demand and case complexity during disruptive events is crucial. Enhancing preventive care and ongoing support could potentially reduce repeated hospitalizations and improve overall patient outcomes in future crises. Furthermore, the pandemic has highlighted the importance of telehealth as a crucial tool for providing continuous care and support during lockdowns and social distancing. While telehealth in EDs has been applied since the beginning with positive effects, it appears challenging to implement for individuals with severe and clinically significant profiles (Raykos et al., 2021; Gorrell et al., 2022), such as those included in this study. Indeed, while telehealth offers flexibility and accessibility, further research is necessary to evaluate its long-term effectiveness in ED treatment, calling for studies on hybrid approaches (Freeman, 2022). On the other hand, telehealth emerged as a crucial tool for maintaining care continuity during lockdowns, but its effectiveness, particularly for severe ED cases, remains debated. A study by Murphy-Morgan et al. (2024) found that while telehealth provided essential social support during isolation, it was not always sufficient for individuals with more complex psychiatric needs. These patients faced barriers such as digital literacy issues and the inability to engage meaningfully in virtual therapy settings. Hybrid care models that combine in-person and remote care were recommended to improve outcomes for these individuals (Monaghesh and Hajizadeh, 2020). The pandemic exposed critical gaps in ED care, particularly the limitations of telehealth for patients with severe illness. While telehealth proved valuable for maintaining some continuity of care during lockdowns, its effectiveness for complex cases was hampered by factors such as digital literacy barriers and the need for in-person interaction (Bouabida et al., 2022).

This necessitates a multi-pronged approach: developing specialized interventions for severe ED cases, conducting further research on optimal telehealth implementation and hybrid models, and implementing policy changes to ensure equitable access to both in-person and remote ED care.

Policies promote telemedicine to reduce ED overcrowding and ensure equitable access to care, especially for vulnerable populations. They also facilitate real-time data sharing for informed decision-making and coordinate healthcare responses with public health measures. By focusing on preparedness, efficient resource use, and workforce support, healthcare policy helps prevent ED strain and ensures a more effective and equitable response during crises (Raykos et al., 2021). Future healthcare policies should aim to integrate mental health crisis management protocols into national emergency preparedness frameworks. This includes strengthening collaborations between public health institutions, mental health professionals, and digital health services to ensure a more seamless response in future crises. Moreover, policies should emphasize reducing disparities in access to ED care by funding initiatives that specifically target lower-income populations, who are disproportionately affected by barriers to mental health services.

## Limitations and future directions

This study has several limitations that warrant acknowledgment. Firstly, its retrospective design may introduce bias, as it relies on historical data that were not originally collected with the current research objectives in mind. Additionally, being a single-center study further limits the generalizability of the findings. The descriptive and naturalistic approach of the study, while valuable for capturing real-world clinical conditions, lacks the rigor and control typically found in experimental studies, which can impact the consistency of treatment and outcome measurement. Our limitation is sampling bias, since the sample is not representative of the entire population.

Furthermore, the sample comprises individuals admitted for inpatient treatment, generally representing those with more severe symptomatology. The study’s exclusive focus on a sample of inpatients with severe EDs significantly restricts the applicability of its findings to the broader population of individuals experiencing EDs. Therefore, while the study provides valuable insights into the experience of this specific, severely affected population, it is crucial to avoid overgeneralizing the findings to all individuals with EDs. The observed trends and insights, particularly regarding the impact of the COVID-19 pandemic, may not be directly transferable to those with milder presentations or different care pathways. Integrated care models that combine medical, psychological, and social support are essential, as EDs often have complex biopsychosocial underpinnings. Establishing multidisciplinary teams that include dietitians, mental health professionals, and social workers can offer holistic care to address both the psychological and physical aspects of EDs. This approach ensures patients receive comprehensive treatment for comorbidities like anxiety, depression, and psychosis, which were heightened during the pandemic. Telehealth services, while beneficial, should be augmented with in-person therapy where possible, especially for patients with severe psychiatric presentations. Implementing hybrid models combining digital and face-to-face consultations can improve access to care, particularly for those in remote or underserved areas. However, it is crucial to enhance the digital literacy of patients and caregivers to ensure meaningful engagement in remote therapy. Given the impact of social isolation on ED symptoms, community-based interventions that foster social connectedness can be particularly effective. Peer support groups, whether online or in person, can provide patients with a sense of belonging and reduce feelings of isolation, a key trigger for disordered eating behaviors. Addressing the socio-economic stressor exacerbated by the pandemic is essential. Access to financial support, educational resources, and vocational training can alleviate the pressures faced by lower-income individuals, who are at higher risk for ED exacerbation during crises. Lastly, public health campaigns that promote body positivity and counter harmful social media messages regarding weight and appearance can help reduce the social pressures that fuel EDs, particularly among adolescents. These multifaceted interventions, designed to address both psychosocial and structural determinants, are crucial for mitigating the long-term impact of global crises on ED patients. Furthermore, as this study was conducted in a residential treatment center, it primarily captures individuals with severe ED symptomatology. This limits the generalizability of our findings to outpatient or community-based ED populations. Future studies should incorporate multicenter datasets and longitudinal designs to assess long-term outcomes and variations in treatment response across different care settings.

## Conclusion

This study reveals how global crises, like the COVID-19 pandemic, exacerbate EDs, particularly among younger individuals and those with lower educational attainment. Pandemic-related socioeconomic and psychological stressors, including reduced access to mental healthcare, financial instability, and disruptions to social and educational support, significantly contributed to the onset or worsening of ED symptoms. The increased incidence of comorbid mental illnesses and psychotic symptoms, coupled with the greater use of antipsychotic medications, underscores the urgent need for adaptable healthcare systems capable of addressing the growing complexity of ED cases during and after crises. Early intervention and targeted mental health services are crucial, especially for young people. While telehealth proved useful during lockdowns, its limitations for complex cases highlight the need for hybrid care models combining in-person and remote services. Longitudinal studies are essential to understand the long-term effects of the pandemic and to optimize treatment approaches for this vulnerable population. These studies should focus on developing age-appropriate interventions that address the unique developmental, educational, and psychosocial challenges faced by younger ED patients, integrating evidence-based practices and diverse support systems while acknowledging the limitations of telehealth in complex cases. Additionally, given the significant impact of interpersonal difficulties on ED pathology, future research should emphasize the role of targeted psychotherapeutic interventions aimed at improving interpersonal relationships and social functioning. Therapies such as Interpersonal Psychotherapy (IPT) and Cognitive Behavioral Therapy (CBT-E) should be further explored to help patients develop healthier coping strategies, enhance emotional regulation, and rebuild supportive social networks. Such research will be critical to developing effective, targeted interventions for young people affected by ED.

## Data Availability

The raw data supporting the conclusions of this article will be made available by the authors, without undue reservation.
